# Procedure‐specific temperature dependence and lag structure of urinary stone surgery: A nationwide open‐data study in Japan, 2019–2024

**DOI:** 10.1002/bco2.70270

**Published:** 2026-08-18

**Authors:** Yuto Sakurai, Takashi Kawahara, Akihito Hashizume, Masanobu Yamazaki, Teppei Takeshima, Shinnosuke Kuroda, Tomoyuki Tatenuma, Hiroji Uemura, Kazuhide Makiyama, Jun‐ichi Teranishi

**Affiliations:** ^1^ Departments of Urology and Renal Transplantation Yokohama City University Medical Center Yokohama Japan; ^2^ Department of Urology Yokohama City University, Graduate School of Medicine Yokohama Japan

**Keywords:** ambient temperature, extracorporeal shock wave lithotripsy, Japan, National Database (NDB), percutaneous nephrolithotomy, seasonality, ureteroscopic lithotripsy, urolithiasis

## Abstract

**Objectives:**

To determine whether the seasonality of urinary stone surgery and its association with ambient temperature differ between surgical procedures and to compare the time lag between temperature and each procedure.

**Subjects and methods:**

We used the nationwide open data of the National Database of health insurance claims in Japan for fiscal years 2019–2024 (April 2019 to March 2025, 72 months). We counted monthly inpatient surgeries for extracorporeal shock wave lithotripsy (ESWL), ureteroscopic lithotripsy (URS), percutaneous nephrolithotomy (PCNL) and transurethral cystolitholapaxy and expressed them as a seasonal index of surgeries per business day normalized to a mean of 1 within each fiscal year. Observed monthly mean temperatures for 12 representative meteorological stations were correlated with the index at lags of −1 to +4 months.

**Results:**

A total of 71 791 ESWL, 320 863 URS, 71 272 cystolitholapaxy and 20 387 PCNL procedures were performed. Volume rose from summer to autumn and was lowest in winter for ESWL, URS and cystolitholapaxy. In the same month, the correlation with temperature was strongest for ESWL (*r* = 0.83, 95% CI 0.74–0.89), followed by URS (*r* = 0.43, 0.22–0.60) and cystolitholapaxy (*r* = 0.35, 0.13–0.54), whereas PCNL showed no association (*r* = 0.05, −0.19–0.27). The correlation peaked at 1 month for ESWL (*r* = 0.87), at 2 months for URS (*r* = 0.69) and cystolitholapaxy (*r* = 0.52) and at 3 months for PCNL, where it was not significant at any lag. PCNL showed significant month‐to‐month variation that was not explained by temperature.

**Conclusion:**

The seasonality of urinary stone surgery and the time lag relative to ambient temperature differ by procedure, being shortest for ESWL and longer for procedures requiring anaesthesia and hospitalization, while PCNL shows no association with temperature.

## 1 INTRODUCTION

Urine volume is an important factor in the development of urinary stones, and a randomized trial has shown that a higher urine volume significantly reduces stone recurrence.^1^ In summer, when the temperature is high, sweating and dehydration concentrate the urine, and this is reported to increase stone onset and hospital visits.^2^ The link between higher ambient temperature and stone prevalence or presentation has been shown consistently around the world. In the United States, climate change is predicted to increase stone prevalence,^3^ and a time‐series study has shown an association between daily mean temperature and the number of stone presentations.^4^

Several studies have reported an association between temperature or season and stone onset.^3^, ^4^, ^5^ However, few large studies have examined how seasonality and temperature sensitivity differ among the surgical procedures used to treat stones. Stone treatment is chosen according to the location, size and composition of the stone and includes ESWL, transurethral lithotripsy (URS), percutaneous nephrolithotomy (PCNL) and transurethral cystolitholapaxy. Whether the association with temperature is the same across these procedures is not clear.

In this study, we used the open data of the National Database (NDB) of health insurance claims in Japan, which covers almost all reimbursed care in the country. This allows us to capture urinary stone surgeries performed in Japan across all procedures and without depending on a single institution or region. The epidemiology of stone treatment in Japan has been reported before,^6^, ^7^ and clinical guidelines are available,^8^ but a nationwide study of seasonality and temperature dependence is still limited.

Japan has a climate with four clear seasons, and in recent years, the summer heat has been especially severe. According to the Fire and Disaster Management Agency, 97 578 people were transported by ambulance for heat‐related illness nationwide in 2024 (May–September), the highest number since the survey began in 2008.^9^ According to the Vital Statistics of the Ministry of Health, Labour and Welfare, 2152 people died from heat‐related illness in 2024.^9^ In such a hot environment, clarifying the seasonality of urinary stones and their association with temperature is important for prevention and for the allocation of medical resources.

## 2 MATERIALS AND METHODS

In this study, we used the open data of the NDB of health insurance claims in Japan, which covers almost all reimbursed care in the country (Ministry of Health, Labour and Welfare, NDB Open Data).^10^ We studied urinary stone surgeries (K‐code procedures) performed during hospitalization from fiscal year 2019 to fiscal year 2024 (April 2019 to March 2025, 72 months in total). The start of the study period was determined by the structure of the data source: Monthly tabulations of surgical procedures are available in the NDB Open Data only from the fiscal year 2019 edition onward, earlier editions providing prefecture‐level rather than monthly breakdowns, and fiscal year 2024 was the most recent edition available at the time of analysis. We analysed the following four procedures for urinary stones: extracorporeal shock wave lithotripsy (ESWL, K768), ureteroscopic lithotripsy (URS, K781, corresponding to transurethral ureterolithotripsy in the Japanese fee schedule; the laser type and the other type were combined), percutaneous nephrolithotomy (PCNL, K764) and transurethral cystolitholapaxy (K798). This study was approved by the ethics committee of Yokohama City University Medical Center, Yokohama, Japan (approval number F260700015). The NDB Open Data are public aggregated data, and individual patients cannot be identified.

We counted the number of surgeries for each procedure by month (6 fiscal years × 12 months = 72 points). Cells that were masked because the count was less than 10 were treated as 0. Most surgeries are performed on weekdays, and the number of weekdays and holidays in a month can affect the count. Therefore, we adjusted the count to the number of surgeries per business day instead of per calendar day. A business day was defined as a weekday that is not a holiday, and holidays were identified with jpholiday, which also accounts for substitute holidays and national holidays. In addition, because the number of surgeries and its trend over time differ among procedures and among fiscal years, we normalized the number of surgeries per business day in each month so that the mean within each fiscal year was 1, and we used this value as the seasonal index (the effect of this adjustment is shown in Figure S1).

For temperature, we used the observed monthly mean temperature for each of the 72 study months, taken as the unweighted mean of 12 representative meteorological stations covering all four climatological regions of Japan (Sapporo, Aomori, Sendai, Tokyo, Niigata, Nagoya, Osaka, Hiroshima, Takamatsu, Fukuoka, Kagoshima and Naha; Japan Meteorological Agency). Observed values were used in preference to climatological normals so that temperature varies between years within the same calendar month; the mean within‐calendar‐month standard deviation was 0.91°C. The mean summer (June to September) temperature increased from 24.9°C in fiscal year 2019 to 26.6°C in fiscal year 2024.

We assessed the difference among months with the Kruskal–Wallis test, and we expressed the size of the seasonality as the ratio of the highest month to the lowest month of the seasonal index (peak‐to‐trough ratio). We assessed the association between the seasonal index and the monthly mean temperature with the Pearson correlation coefficient, and we calculated the 95% confidence interval with the Fisher z transformation. We obtained the change per 1°C as the slope of the seasonal index regressed on temperature, and we tested whether the strength of the correlation differed among procedures with the Williams test for dependent correlations that share the same temperature variable using URS as the reference. Because the 72 monthly observations are clustered within calendar months, we repeated the analysis using month‐clustered robust standard errors and using the 12 calendar‐month means as a conservative alternative, and we additionally fitted a model with calendar‐month fixed effects to isolate the within‐month temperature effect.

Because there can be a time lag from heat exposure to stone formation, presentation and surgery, we shifted the temperature by −1 to +4 months relative to the surgery month and calculated the correlation at each lag. This window was pre‐specified: Previous time‐series studies report that stone‐related presentations rise within days to a few weeks of high ambient temperature, while the intervals for stone formation, presentation and scheduling of surgery are additive and extend the plausible interval to a small number of months, so +4 months was chosen to lie beyond any physiologically plausible interval. A lag of −1 month was included as a negative‐control lag, since temperature occurring after an operation cannot plausibly influence it. The lag with the highest correlation was defined as the peak lag of each procedure; because this is selected as the best of six candidate lags, the correlation at the peak lag is expected to be biased upward and is reported with that caveat. A distributed‐lag model including lags 0–3 simultaneously was fitted as a sensitivity analysis only, as it is under‐powered with 12 monthly means and four collinear terms. We used Python (pandas, NumPy, SciPy, statsmodels, matplotlib, jpholiday) for the analysis. The authors used Claude (Anthropic, San Francisco, CA, USA) to assist with writing the code used to generate the figures and with editing and proofreading the English text. The authors reviewed and verified all output and take full responsibility for the content of this manuscript.

## 3 RESULTS

**FIGURE 1:**
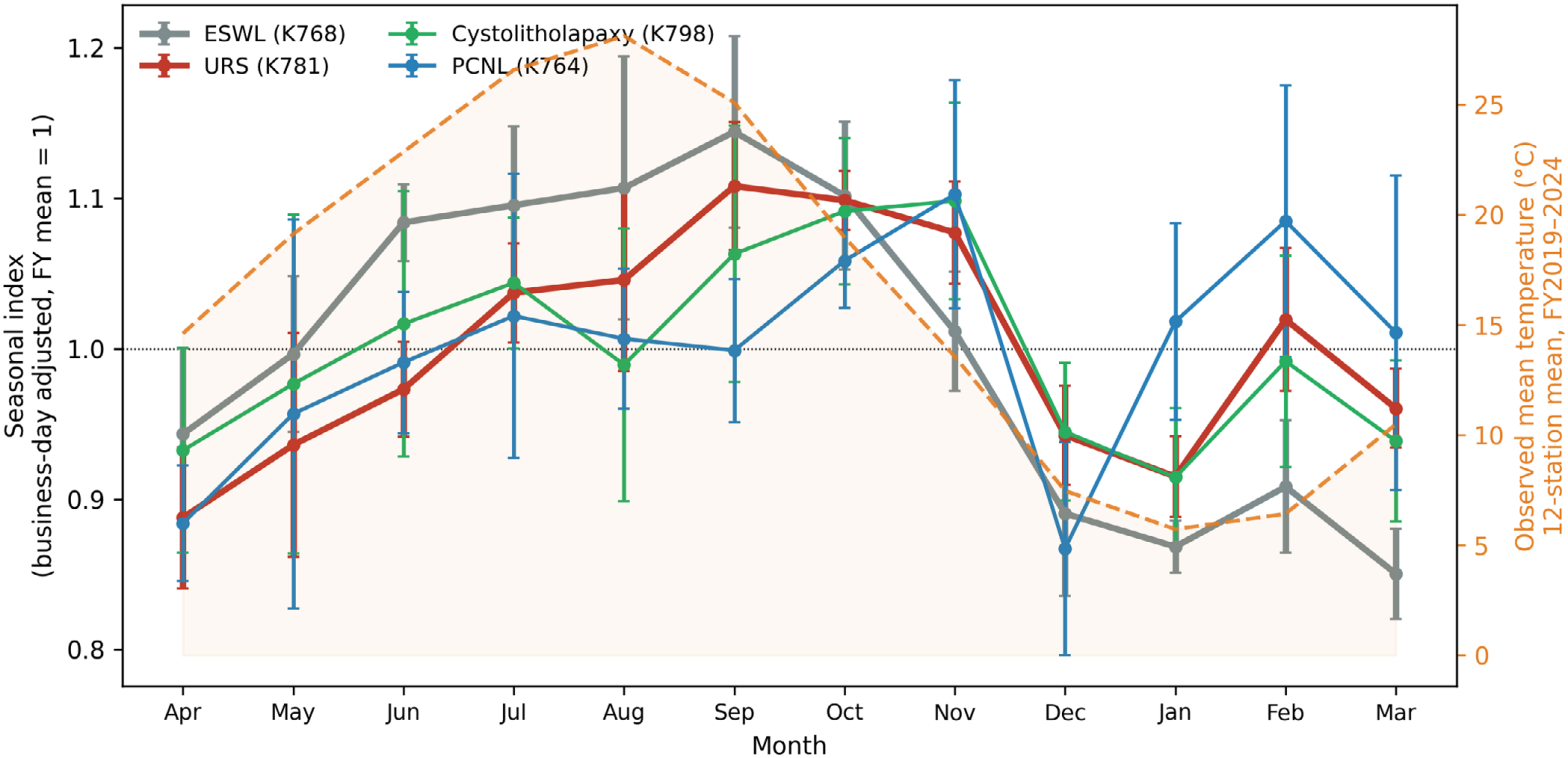
Monthly seasonal index of each surgery and observed mean monthly temperature. The seasonal index is the number of surgeries per business day, normalized so that the mean within each fiscal year is 1. Points are means across the six fiscal years with 95% confidence intervals. The dashed line shows the observed mean monthly temperature averaged over 12 meteorological stations for fiscal years 2019 to 2024. ESWL, extracorporeal shock wave lithotripsy; PCNL, percutaneous nephrolithotomy; URS, ureteroscopic lithotripsy.

During the study period (April 2019 to March 2025), a total of 71 791 ESWL, 320 863 URS, 71 272 transurethral cystolitholapaxy and 20 387 PCNL procedures were performed (Table 1). We compared the monthly business‐day‐adjusted seasonal index of each procedure with the observed mean monthly temperature (Figure 1). For ESWL, URS and cystolitholapaxy, the number of surgeries increased from summer through autumn and was lowest in winter and early spring, with the seasonal index peaking between September and November and reaching its trough between December and April. PCNL did not follow this pattern. The same seasonal pattern was reproduced in every fiscal year (Figure S2). Monthly variation was statistically significant for all four procedures (Kruskal–Wallis, *p* < 0.001), and the seasonal amplitude was largest for ESWL (peak‐to‐trough ratio 1.35) and 1.20–1.27 for the other procedures.

**TABLE 1:** Seasonality and temperature dependence of urinary stone surgery, by procedure.

Procedure	Total *n*	Peak/trough month	Peak‐to‐trough ratio	Kruskal–Wallis *p*	r with temperature, same month (95% CI)	Slope, %/°C (95% CI)	Peak lag, months (*r*)	Williams *p* versus URS
ESWL (K768)	71 791	Sep/Mar	1.35	<0.001	0.83 (0.74–0.89)	+1.18 (0.99–1.37)	1 (0.87)	<0.001
URS (K781)	320 863	Sep/Apr	1.25	<0.001	0.43 (0.22–0.60)	+0.45 (0.22–0.67)	2 (0.69)	—
Cystolitholapaxy (K798)	71 272	Nov/Jan	1.20	<0.001	0.35 (0.13–0.54)	+0.39 (0.14–0.64)	2 (0.52)	0.232
PCNL (K764)	20 387	Nov/Dec	1.27	<0.001	0.05 (−0.19–0.27)	+0.06 (−0.24–0.35)	3 (0.22)	<0.001

*Note*: Seasonal index = number of surgeries per business day, normalized so that the mean within each fiscal year is 1. Correlation and slope are against the observed monthly mean temperature, taken as the unweighted mean of 12 representative meteorological stations (Japan Meteorological Agency). Peak lag = the lag (in months) at which the correlation is highest among the six candidate lags examined (−1 to +4 months); because it is selected as the best of six, the correlation at the peak lag is expected to be biased upward. The Williams test compares the strength of the correlation with that of URS. NDB Open Data, inpatient surgeries, fiscal years 2019–2024 (72 months).

Abbreviations: ESWL, extracorporeal shock wave lithotripsy; PCNL, percutaneous nephrolithotomy; URS, ureteroscopic lithotripsy.

**FIGURE 2:**
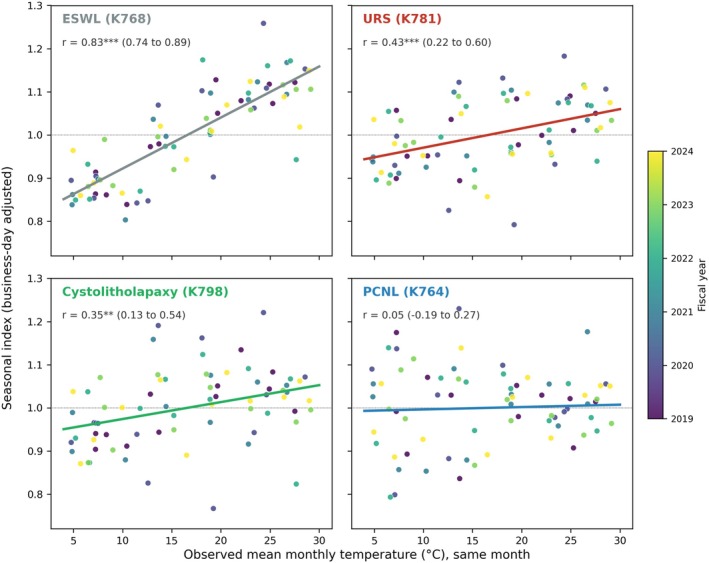
Seasonal index plotted against the observed mean temperature of the same month, for each procedure. Each point is one of the 72 study months and is coloured by fiscal year. Lines are linear fits. *r* is the Pearson correlation with its 95% confidence interval. **p < 0.01; ***p < 0.001.

In the same month, the correlation between the seasonal index and mean monthly temperature was strongest for ESWL (*r* = 0.83, 95% CI 0.74–0.89), followed by URS (*r* = 0.43, 0.22–0.60) and cystolitholapaxy (*r* = 0.35, 0.13–0.54), whereas PCNL showed no association (*r* = 0.05, −0.19–0.27) (Figure 2). The temperature sensitivity, expressed as the slope of the seasonal index on temperature, was +1.18%/°C for ESWL, +0.45%/°C for URS, +0.39%/°C for cystolitholapaxy and +0.06%/°C for PCNL. The correlation for URS differed significantly from that for ESWL and for PCNL (Williams test, both *p* < 0.001) but not from that for cystolitholapaxy (*p* = 0.23). With month‐clustered robust standard errors, the slope remained significant for ESWL (*p* < 0.001), URS (*p* = 0.007) and cystolitholapaxy (*p* = 0.008) but not for PCNL (*p* = 0.81). In the more conservative analysis using the 12 calendar‐month means, the same‐month correlation was significant for ESWL (*r* = 0.91, *p* < 0.001) but not for URS (*r* = 0.49, *p* = 0.11) or cystolitholapaxy (*r* = 0.51, *p* = 0.09). All sensitivity analyses are summarized in Table S2, and the 12‐calendar‐month‐mean analysis is shown in Figure S3.

**FIGURE 3:**
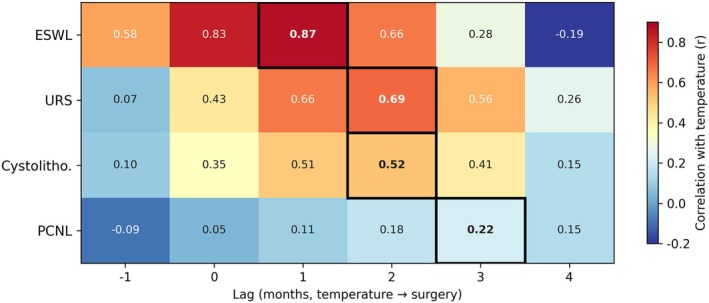
Correlation between temperature and the seasonal index at lags of −1 to +4 months. Colour shows the Pearson correlation coefficient. The black box marks the lag with the highest correlation for each procedure. A lag of −1 month is a negative‐control lag. Numerical values with significance markers are given in Table S1.

**FIGURE 4:**
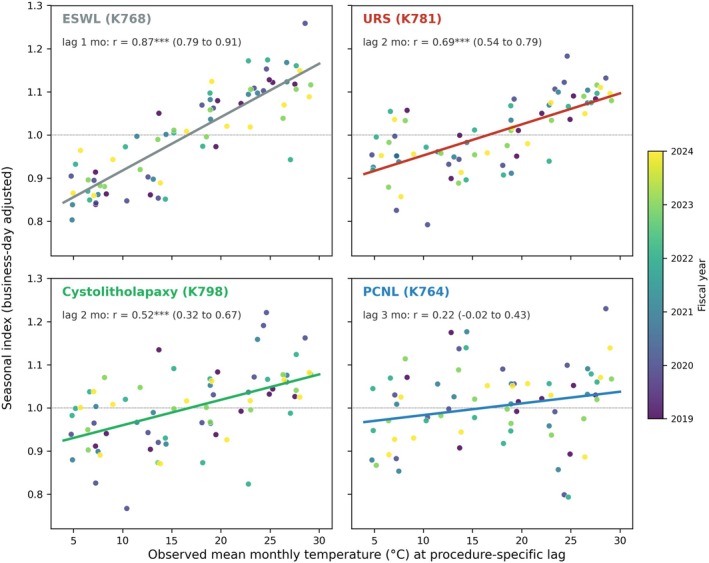
Seasonal index plotted against the observed mean temperature at each procedure's peak lag (1 month for ESWL, 2 months for URS and cystolitholapaxy, 3 months for PCNL). Each point is one study month and is coloured by fiscal year. Because the peak lag is selected as the lag with the highest correlation among six candidates, the correlation shown is expected to be biased upward. ***p < 0.001.

We next examined the lag structure of the temperature–surgery association by shifting temperature from −1 to +4 months relative to the surgery month (Figure 3 and Table S1). The correlation peaked at the shortest lag for ESWL (1 month, *r* = 0.87, 95% CI 0.79–0.91) and at longer lags for URS and cystolitholapaxy (both 2 months, *r* = 0.69, 0.54–0.79 and *r* = 0.52, 0.32–0.67, respectively), all of which remained significant with month‐clustered robust standard errors (all *p* < 0.001) and in the 12‐month‐mean analysis (*r* = 0.95, *p* < 0.001; *r* = 0.79, *p* = 0.002; *r* = 0.77, *p* = 0.004, respectively) (Figure 4). For PCNL, the highest correlation occurred at a lag of 3 months but was not significant (*r* = 0.22, −0.02–0.43; clustered *p* = 0.32; 12‐month‐mean *r* = 0.33, *p* = 0.30), and PCNL was not associated with temperature at any lag examined. The lag profiles were smooth and single‐peaked for every procedure. Because the peak lag is selected as the best of six candidate lags, the correlation at the peak lag is expected to be biased upward; the ordinal pattern of peak lags, rather than the magnitude of the lag‐aligned correlation, is the finding we emphasize.

## 4 DISCUSSION

In this study, we used a comprehensive surgical database in Japan to examine the association between temperature and urinary stone surgery by procedure. ESWL had the strongest correlation with temperature, and URS also correlated with temperature but with a later peak lag than ESWL (the peak lag was 1 month for ESWL and 2 months for URS). The procedure for a urinary stone is chosen according to the location, size and composition of the stone.^8^ ESWL is a low‐invasive treatment that needs little or no anaesthesia and can be done in an outpatient setting, whereas URS (transurethral surgery) is an inpatient surgery performed in the operating room under anaesthesia, so the two differ greatly in invasiveness and in how they are arranged.^11^ Because of this difference, ESWL can be performed quickly and early after onset, while URS needs time for preparation and scheduling between the hospital visit and the surgery, and we think this appeared as the 1‐month delay. In contrast, PCNL showed no association with ambient temperature, although its month‐to‐month variation in volume was statistically significant. The target of PCNL is the kidney rather than the ureter,^8^ so the gap between symptom onset and the time of treatment is large, and kidney stones may be less affected by temperature in their onset; these may be the reasons for the lack of a temperature association. The significant month‐to‐month variation in PCNL volume is more likely to reflect non‐thermal factors such as the scheduling and staffing constraints of a complex inpatient procedure, referral and case‐mix patterns and accumulated case backlog, none of which can be distinguished using aggregated open data. The finding that PCNL was not associated with temperature at any lag examined can be interpreted as a negative control, which supports the idea that the associations we found are specific to heat‐sensitive stones and procedures rather than reflecting seasonal variation common to all surgery. We emphasize that this refers to the absence of a temperature association and not to an absence of seasonal variation in PCNL volume.

A feature of this study is that we analysed the number of surgeries rather than the diagnosis. In some databases such as MDV, the diagnosis can change, for example, because of switching of insurance, so it can be difficult to count patients exactly. Because this study used the number of surgeries that were actually billed, it is less affected by this problem. Many previous studies, in Japan and other countries, have used emergency department visits for renal colic,^12^ hospitalizations for stones^13^ or visit and onset events^4^, ^5^ as the unit and have assessed the association with temperature or climate. These reflect the dynamics of onset or visits, but they do not always reflect the dynamics of the treatment that was actually performed. By using the number of surgeries as the unit, as in this study, we can directly capture the seasonality of stone care that was actually delivered, by procedure. We have also worked on clinical studies of stone care, such as predicting the composition of ureteral stones^14^ and a preoperative model for ureteral length for ureteral stent placement,^15^ but this is the first time we examined the association with seasonality and temperature on a nationwide scale.

Previous studies have reported that the lag from a rise in temperature to a stone‐related visit is relatively short, from a few days to a few weeks.^4^ A short‐term association between climate or temperature and emergency visits for renal and urinary disease has also been shown.^16^ In contrast, in this study the peak of the number of surgeries appeared 1–2 months after the temperature. This difference shows that the timing of onset and visit is different from the timing of treatment (surgery), and for procedures that need anaesthesia and hospitalization, the interval from the visit to the surgery is added as an extra lag. This view may also have practical implications for the allocation of medical resources, such as securing operating‐room and ESWL slots in anticipation of the increase in surgical demand from summer to early autumn.

Our findings can be placed alongside international work on the resource and economic consequences of climate‐related stone disease. In the United States, a warming climate has been projected to expand the stone belt and to increase both the prevalence of urolithiasis and the associated treatment costs [PMID: 18626008], and independent projections that account for population growth and the rising prevalence of obesity and diabetes estimate an additional 1.24 billion US dollars per year in the cost of stone disease by 2030 [PMID: 25015037], against an annual cost already estimated in the billions of dollars in earlier national assessments [PMID: 15711292]. Reviews of environmental determinants likewise identify relatively high temperature and low humidity as conditions that favour stone formation across geographical settings [PMID: 40029430], and a recent scoping review of the effects of climate change on surgery highlights how little is known about the consequences for surgical service delivery [PMID: 41646046]. Almost all of this literature is framed in terms of disease prevalence, presentations or aggregate cost. Our results add the operational dimension that is missing from those projections: If the demand that reaches the operating room follows ambient temperature with a procedure‐specific delay of 1–2 months, then the same warming that is projected to raise prevalence will also shift when capacity is needed, and differently for day‐case and inpatient procedures. Whether the lag structure we observed in Japan is reproduced in other health systems, in which access, referral routes and the balance between ESWL and ureteroscopy differ, remains to be tested.

The relationship between stone composition and seasonality is a further question that our data cannot address, because stone composition is not recorded in the NDB Open Data. The available evidence suggests that it is unlikely to be uniform across stone types. In Germany, uric acid stone formers presented with renal colic significantly more often in the third and fourth quarters of the year, whereas no seasonal variation was found in calcium oxalate stone formers in the same region; notably, the authors concluded that temperature alone was not a sufficient explanation, since the two quarters concerned span both a warm and a cold season [PMID: 35690647]. That conclusion parallels our own finding that the association we observed is carried by the seasonal cycle rather than by year‐to‐year temperature variation. Because low urine volume and low urine pH favour uric acid crystallization, a composition‐specific analysis is biologically plausible and would help to separate a genuinely thermal mechanism from other seasonal influences. This will require data sources that link stone analysis to the date of treatment.

This study has several limitations. First, because this study is based on a database of surgical procedures, we cannot know the size, location and composition of the stones or detailed clinical information about the patients. The interval from presentation to surgery is also not recorded, so our interpretation that the longer lag for URS reflects the time needed to schedule inpatient surgery under anaesthesia is a hypothesis and was not measured in this study. Second, the data do not allow a regional analysis of surgical volume. In the NDB Open Data for fiscal year 2019 onward, the surgical tables are tabulated by month, whereas prefecture‐level tabulations are provided in separate tables that cannot be cross‐classified by month. This matters because the amplitude of the seasonal exposure differs markedly within Japan: In our temperature data, the annual range of monthly mean temperature was 11.9°C in Okinawa but 25.3°C in northern Japan, and the warmest month in Okinawa was July rather than August. The timing of the seasonal cycle was nevertheless almost identical across regions (correlation with the national profile *r* > 0.99 for every region), so we consider a nationwide analysis of seasonal timing to be interpretable, while regional heterogeneity in amplitude cannot be resolved with these data.

Third, and most importantly for interpretation, the association we observed is driven by between‐month (seasonal) variation rather than by year‐to‐year variation in temperature. When we added calendar‐month fixed effects, so that the estimate reflects only whether a given month was hotter than usual in a given year, the temperature effect was not significant for any procedure (ESWL −0.07%/°C, 95% CI −1.38 to +1.23; URS + 0.10%, −0.99 to +1.19; cystolitholapaxy −0.17%, −2.00 to +1.66; PCNL +1.42%, −0.49 to +3.33). This analysis is strongly under‐powered, because the within‐month standard deviation of temperature was only 0.91°C compared with a seasonal range of approximately 22°C, and the confidence intervals include the seasonal estimates. It therefore neither refutes nor independently confirms the association, but it does mean that ambient temperature cannot be separated from other factors that co‐vary with season, such as fluid intake behaviour, holiday and vacation scheduling and referral patterns. Our findings should accordingly be read as a seasonal association that is consistent with a temperature‐related mechanism rather than as evidence that temperature itself drives surgical volume.

Finally, temperature was represented by the unweighted mean of 12 representative meteorological stations and was not weighted by population, the peak lag for each procedure was selected as the lag with the highest correlation among six candidate lags and the correlation at that lag is therefore expected to be biased upward, this study is an ecological analysis based on monthly aggregation and does not demonstrate the causal path from onset to surgery in individual patients, and because cells masked at counts below 10 were treated as 0, a small error may occur for procedures with few cases.

## 5 CONCLUSION

This study showed that the seasonality of urinary stone surgery in Japan and its association with ambient temperature differ by procedure. ESWL showed the strongest association with temperature and the shortest lag of 1 month, whereas URS and cystolitholapaxy peaked at 2 months, consistent with the additional time required to schedule inpatient surgery under anaesthesia. PCNL showed significant month‐to‐month variation but no association with ambient temperature at any lag examined.

## AUTHOR CONTRIBUTIONS

YS: Data curation; formal analysis; writing—original draft. TK: Conceptualization; supervision; writing—review and editing. AH, MY, TT, and SK: Investigation; writing—review and editing. All authors: Final approval of the manuscript.

## CONFLICT OF INTEREST STATEMENT

We declare no conflicts of interest.

## Supporting information


**Figure S1.** Comparison of calendar‐day and business‐day adjustment. Left: URS seasonal index by both methods. Right: correlation with temperature for each procedure by both methods.

**Figure S2.** URS seasonal index for each fiscal year (2019 to 2024). The pattern was similar every year. Fiscal year 2020 was lower in the first half during the COVID‐19 period.

**Figure S3.** Seasonal index plotted against temperature using the 12 calendar‐month means, the most conservative analysis. Points are means with 95% confidence intervals and numbers indicate the month. Compare with Figure 2, which uses all 72 monthly observations.

**Table S1.** Correlation between temperature and the seasonal index at every lag examined, with significance markers.

**Table S2.** Sensitivity analyses accounting for the clustered structure of the data, including month‐clustered robust standard errors, the 12‐calendar‐month‐mean analysis, and the within‐month temperature effect from a model with calendar‐month fixed effects.

## Data Availability

The data that support the findings of this study are available in the Supporting Information of this article.
