# Supplementary material for: Procedure‐specific temperature dependence and lag structure of urinary stone surgery: A nationwide open‐data study in Japan, 2019–2024
Source: BJUI Compass. 2026 Aug 18;7(8):e70270. doi: 10.1002/bco2.70270 (PMC13483297; doi:10.1002/bco2.70270)
Supplement: Supplementary file 1 [file BCO2-7-e70270-s002.docx]

**Supplementary Table S2. Sensitivity analyses accounting for the clustered structure of the data**

| **Procedure** | **Same month: clustered p** | **Same month: 12-month means, r (p)** | **Peak lag: clustered p** | **Peak lag: 12-month means, r (p)** | **Within-month effect, %/°C (95% CI)** | **Within-month p** |
| --- | --- | --- | --- | --- | --- | --- |
| **ESWL** | <0.001 | 0.91 (<0.001) | <0.001 | 0.95 (<0.001) | -0.07 (-1.38 to +1.23) | 0.910 |
| **URS** | 0.007 | 0.49 (0.108) | <0.001 | 0.79 (0.002) | +0.10 (-0.99 to +1.19) | 0.852 |
| **Cystolitholapaxy** | 0.008 | 0.51 (0.090) | <0.001 | 0.77 (0.003) | -0.17 (-2.00 to +1.66) | 0.857 |
| **PCNL** | 0.810 | 0.04 (0.896) | 0.322 | 0.33 (0.296) | +1.42 (-0.49 to +3.33) | 0.143 |

Clustered p = p value for the slope on temperature using robust standard errors clustered by calendar month (n = 72). 12-month means = Pearson correlation computed on the 12 calendar-month means (n = 12), the most conservative approach. Within-month effect = slope on temperature in a model that also includes calendar-month fixed effects, which isolates the effect of a given month being hotter than usual in a given year; this analysis is strongly under-powered because the within-month standard deviation of temperature was 0.91 °C compared with a seasonal range of approximately 22 °C. Peak lag is 1 month for ESWL, 2 months for URS and cystolitholapaxy, and 3 months for PCNL. URS, ureteroscopic lithotripsy; ESWL, extracorporeal shock wave lithotripsy; PCNL, percutaneous nephrolithotomy.
