# Supplementary material for: Procedure‐specific temperature dependence and lag structure of urinary stone surgery: A nationwide open‐data study in Japan, 2019–2024
Source: BJUI Compass. 2026 Aug 18;7(8):e70270. doi: 10.1002/bco2.70270 (PMC13483297; doi:10.1002/bco2.70270)
Supplement: Supplementary file 2 [file BCO2-7-e70270-s004.docx]

**Supplementary Table S1. Correlation between temperature and the seasonal index at every lag examined**

| **Procedure** | **−1** | **0** | **+1** | **+2** | **+3** | **+4** |
| --- | --- | --- | --- | --- | --- | --- |
| **ESWL** | 0.58* | 0.83* | 0.87* | 0.66* | 0.28* | -0.19 |
| **URS** | 0.07 | 0.43* | 0.66* | 0.69* | 0.56* | 0.26* |
| **Cystolitholapaxy** | 0.10 | 0.35* | 0.51* | 0.52* | 0.41* | 0.15 |
| **PCNL** | -0.09 | 0.05 | 0.11 | 0.18 | 0.22 | 0.15 |

Values are Pearson correlation coefficients between the observed monthly mean temperature at the stated lag (in months, temperature → surgery) and the business-day-adjusted seasonal index, based on all 72 monthly observations. *p < 0.05. A lag of −1 month was included as a negative-control lag. The profiles are smooth and single-peaked for every procedure. URS, ureteroscopic lithotripsy; ESWL, extracorporeal shock wave lithotripsy; PCNL, percutaneous nephrolithotomy.
